# Fabrication and evolution of multilayer silver nanofilms for surface-enhanced Raman scattering sensing of arsenate

**DOI:** 10.1186/1556-276X-6-263

**Published:** 2011-03-28

**Authors:** Jumin Hao, Mei-Juan Han, Zhonghou Xu, Jinwei Li, Xiaoguang Meng

**Affiliations:** 1 Center for Environmental Systems, Stevens Institute of Technology, Hoboken, NJ 07030, USA; 2 Department of Mechanical Engineering, Stevens Institute of Technology, Hoboken, NJ 07030, USA

## Abstract

Surface-enhanced Raman scattering (SERS) has recently been investigated extensively for chemical and biomolecular sensing. Multilayer silver (Ag) nanofilms deposited on glass slides by a simple electroless deposition process have been fabricated as active substrates (Ag/GL substrates) for arsenate SERS sensing. The nanostructures and layer characteristics of the multilayer Ag films could be tuned by varying the concentrations of reactants (AgNO_3_/BuNH_2_) and reaction time. A Ag nanoparticles (AgNPs) double-layer was formed by directly reducing Ag^+ ^ions on the glass surfaces, while a top layer (3rd-layer) of Ag dendrites was deposited on the double-layer by self-assembling AgNPs or AgNPs aggregates which had already formed in the suspension. The SERS spectra of arsenate showed that characteristic SERS bands of arsenate appear at approximately 780 and 420 cm^-1^, and the former possesses higher SERS intensity. By comparing the peak heights of the approximately 780 cm^-1 ^band of the SERS spectra, the optimal Ag/GL substrate has been obtained for the most sensitive SERS sensing of arsenate. Using this optimal substrate, the limit of detection (LOD) of arsenate was determined to be approximately 5 μg·l^-1^.

## Introduction

Since the discovery of surface-enhanced Raman scattering (SERS) in the late 1970s, SERS has been extensively studied as a sensitive analytical technique for fundamental studies of surface species [1-6]. The development of SERS substrates with high sensitivity and good reproducibility has been one of the most challenging tasks. Colloidal Ag or Au nanoparticles are the most widely used SERS substrates. The aggregation of the colloidal particles facilitating the formation of "hotspots" appears to be crucial for strong SERS enhancement [7-11]. However, the aggregation of colloidal particles is difficult to control, thus leading to poor reproducibility of both substrates and SERS signals [12,13]. Although the immobilization/assembly of colloidal nanoparticles onto solid supports could improve the controllable aggregation of the nanoparticles to some extent, the synthesis and fabrication processes for such assembled layers are usually laborious and time consuming, and usually require the use of organic molecules acting as reductants, stabilizing reagents, or coupling reagents.

In recent years, extensive efforts have been dedicated to developing stable nanostructured Ag or Au surfaces directly on solid substrates using various techniques including vacuum evaporation [14], sputtering [15], electrochemical deposition [16], thermal decomposition [17], and electroless plating [18-20]. The electroless plating of nanostructured metal films is attracting much attention due to its easy production, uniform coating, low cost, and no need for special and expensive equipments. A galvanic displacement reaction is a simple electroless plating process to prepare SERS-active Ag or Au films on metal and semiconductive substrates like copper, germanium, and silicon [19-22]. However, it cannot be applied to dielectric substrates like cheap glass slides. Although the well-known mirror reaction is suitable for the deposition of Ag nanofilms onto glass surfaces, this process includes multi-step reactions and requires complex reagents, resulting in difficulty in controlling the surface roughness of the resulting Ag films [20,23,24].

SERS-based techniques have been widely applied to chemical, biological, and medical sensing because SERS has been believed to be one of the most sensitive spectroscopic methods [1,2,5,10,25-27]. Most recently, SERS technique for environmental analysis and monitoring has been reviewed by Halvorson and Vikesland [25], and Alvarez-Puebla and Liz-Marzan [27], respectively. The SERS detections of some inorganic environmental pollutants such as perchlorate (ClO_4_^-^) [21,28,29], arsenate (AsO_4_^3-^) [23], chromate (CrO_4_^2-^) [30], uranyl (UO_2_^2+^) [31,32], cyanide (CN^-^) [30], and thiocyanate (SCN^-^) [33] have been investigated. Arsenic (As) is one of the most toxic contaminants found in the environment, and long-term exposure to arsenic can cause various cancers and other serious diseases [34,35]. Based on the World Health Organization (WHO) guideline, many countries including the US have promulgated a more stringent drinking water standard for arsenic with a maximum contaminant level (MCL) of 10 μg·l^-1 ^(ppb) [35,36]. There exists an urgent need for the development of methods for effective monitoring and measurement of arsenic in the field [37,38].

Currently, the commonly used laboratory methods such as atomic fluorescence spectroscopy (AFS), atomic absorption spectroscopy (AAS), inductively coupled plasma-atomic emission spectrometry or mass spectrometry (ICP-AES or ICP-MS) allow the detection of low arsenic concentration, but they are expensive, bulky, and usually involved in sophisticated and time-consuming preparations of the samples, making them infeasible for field assays. Moreover, these techniques cannot distinguish different arsenic species, such as arsenite (As(III)) and arsenate (As(V)), without sample pretreatments. In this case, the SERS technique, which can be used in conjunction with commercially available portable Raman systems, has emerged as a potentially promising solution in field assays due to its ability to provide ultrasensitive, reliable, non-invasive, nondestructive, fast, simple, and cost-effective measurements. It has been demonstrated that SERS technique is able to identify, detect, and screen single and multiple contaminants simultaneously in a small volume of sample [25,27]. More significantly, it is incomparable in speciation analysis including distinguishing among the arsenic species with no need for any complex sample preparation because it can provide a nice "fingerprint" of materials of interest [38]. The first SERS spectrum of arsenate at high concentrations (> 100 mg·l^-1^) was reported by Greaves and Griffith [39] using silver sols. Recently, Mulvihill et al. [38] fabricated Langmuir-Blodgett (LB) monolayers of polyhedral Ag nanocrystals for arsenate SERS detection in groundwater samples with low concentrations (< 10 μg·l^-1^). Most recently, we examined the effect of ions on the arsenate SERS sensing using Ag nanofilms prepared by modified mirror reaction [23].

In this article, a controllable one-step electroless plating process was applied to directly deposit multilayer Ag nanofilms on glass slides (Ag/GL substrates) for effective SERS sensing of arsenate. The Ag/GL substrates prepared under different conditions were characterized by SEM and UV-Vis spectra, and the formation mechanisms of the multilayer films were discussed. The SERS spectra of arsenate on Ag/GL substrates were analyzed. The relation between the preparation conditions, the resulting morphology of the Ag nanofilms, and the SERS sensitivity to arsenate was examined to optimize the Ag nanofilms for arsenate SERS sensing. Using optimized substrates, the limit of detection of arsenate was determined.

## Experimental

### Materials

Sodium arsenate (Na_3_AsO_4_·7H_2_O) and silver nitrate (AgNO_3_) were purchased from Fisher Scientific (Fair Lawn, NJ, USA). Butylamine (BuNH_2_) was obtained from Sigma-Aldrich (Milwaukee, WI, USA). Anhydrous ethanol (Pharmco-AAPER, Brookfield, CT) was used as a plating solvent. All other chemicals were analytical grade and purchased from Sigma-Aldrich or Fisher Scientific and used as received. Deionized (DI) water with a resistivity of 18.2 MΩ·cm (Millipore Milli-Q System) was used throughout the experiments. Aqueous arsenate samples in the concentration range 0-300 μg·l^-1 ^were prepared by diluting a stock solution of 10^6 ^μg·l^-1 ^with DI water.

### Preparation of Ag nanofilms

The Ag films deposited on glass slides were prepared by reduction of AgNO_3 _with BuNH_2 _in anhydrous ethanol using 6-well plates as reaction vessels, without stirring at room temperature (approximately 22°C). The glass slides were cut into 1 × 1 cm^2^, which were cleaned in piranha solution (a mixture of concentrated H_2_SO_4 _and H_2_O_2 _(30%) with a volume ratio of 70 to 30) at approximately 80°C for 1 h. After being washed with DI water, these glass slides were sonicated in 1 M NaOH solution for 30 min to obtain negatively charged surfaces for improved Ag^+ ^adsorption on them during the AgNPs deposition followed by washing with DI water and ethanol in sequence, and then dried under a stream of compressed nitrogen gas. The cleaned glass slides were put into the wells of the 6-well plates (three slides in each well/batch) and a 14 ml fresh prepared ethanolic AgNO_3_/BuNH_2 _solution was added into each well within 10 min. The concentrations of AgNO_3_/BuNH_2 _in ethanol used in the experiments were 1/0.5 mM, 5/2.5 mM, and 10/5 mM (a constant ratio of 2:1). After a period of reaction time (0.5 to 40 h), one batch of Ag/GL substrates were rinsed with ethanol and dried under a stream of compressed nitrogen gas. Here, all the Ag/GL substrates are labeled as Ag/GL-c/c-t, where "c/c" and "t" represent the concentrations of the AgNO_3_/BuNH_2 _ethanolic solution in "mM" and the reaction time in "hours" or "h", respectively. For example, Ag/GL-5/2.5-18 means an Ag/GL substrate which is prepared in the ethanolic solution containing 5 mM AgNO_3 _and 2.5 mM BuNH_2 _with the reaction time of 18 h.

### Instruments and methods

The morphology and microstructure of the Ag/GL substrates were examined using a field-emission scanning electron microscope (FESEM) (LEO 982, LEO Electron Microscopy Inc., Thornwood, NY, USA) operated at an accelerating voltage of 5 kV and a working distance (WD) of 6 mm. UV-Vis absorbance spectra were recorded with a Synergy™ HT Multi-Detection Microplate Reader (BioTek Instruments Inc., Winooski, VT, USA) with 2 nm resolution in the wavelength range between 350 and 750 nm, and a cleaned glass slide was used as a background. For SERS analysis, 10 μl of arsenate solution was dropped onto the Ag/GL substrate and a sample droplet with a diameter of approximately 3 mm was formed. After air-dried, the SERS spectra were collected in high resolution mode on a Thermo Nicolet Almega XR Dispersive Raman Spectrometer (Thermo Fisher Scientific Inc., Madison, WI, USA) equipped with a CCD detector, an optical microscope and a digital camera, and a 780 nm laser line with a laser source power of 30 mW (50% power was applied in the experiments). The Raman band of a silicon wafer at 520 cm^-1 ^was used to calibrate the spectrometer. All the measurements were conducted in the backscattering geometry. A 10 × microscope objective was used, providing a laser spot size of 3.1 μm. The data acquisition time was 3 s per scan and five scans were used for each spectrum collection. For reliable and reproducible SERS measurements, a mapping method was employed and an averaged spectrum was obtained by averaging 25 spectra splitted from a mapping result with a scan area of 2 × 2 mm^2^, a step size of 0.5 × 0.5 mm^2 ^(*X *× *Y*).

## Results and discussion

### Characterization and evolution process of Ag nanofilms

**Figure 1:**
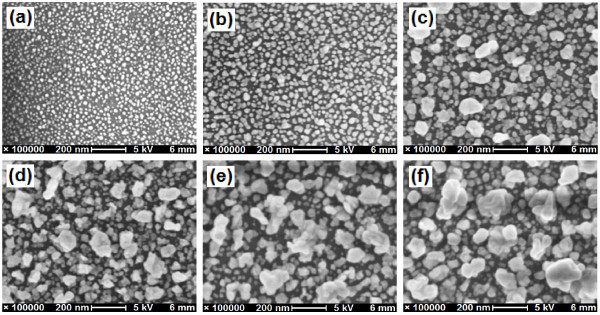
**SEM images of AgNPs multilayer films on glass slides prepared in a AgNO_3_/BuNH_2 _(5/2.5 mM) ethanolic solution with different reaction times**: **(a) **1.5 h, **(b) **2 h, **(c) **5 h, **(d) **18 h, **(e) **25 h, and **(f) **40 h.

To investigate the evolution of the nanostructured Ag films and the relationship between the Ag film structures and the SERS effect, the surface morphologies of the Ag/GL substrates were characterized by SEM observation. Figure 1 presents typical SEM images of the Ag/GL substrates prepared in a 5/2.5 mM AgNO_3_/BuNH_2 _solution at different reaction times of 1.5-40 h. At the beginning stage of the reaction (≤ 2 h), a thin monolayer (1st-layer) consisting of small sphere-like AgNPs formed on the glass surfaces as shown in Figure 1a,b. Most of AgNPs in the monolayer were isolated from their nearest neighbors for the 1.5 h reaction time (Figure 1a). As the reaction time increased to 2 h, the AgNPs grew and some of them appeared to contact each other, and the AgNPs monolayer exhibited a close-packed structure.

By continuously raising the reaction time to 5-40 h, newly reduced Ag atoms would grow on the top of the well-defined 1st-layer AgNPs to form second layer AgNPs (2nd-layer) as shown in Figure 1c,d,e,f. The 2nd-layer AgNPs were much larger than those in the 1st-layer and kept growing in particle size and coverage degree as the reaction time was prolonged. When a growing AgNP met another growing AgNP, coalescence of AgNPs occurred, which led to aggregated and agglomerated AgNPs congeries with irregular shapes and structures (Figure 1d,e,f). Figure 1e,f also indicate that the growth of the 2nd-layer AgNPs in the direction normal to the substrate surface was notable. It was also observed that the growth of the 1st-layer AgNPs was depressed when the reaction time was > 5 h probably due to the formation of the 2nd-layer AgNPs on its top and the competition between the two layers. It is also likely that the formation of the 2nd-layer AgNPs partially consumed the 1st-layer AgNPs through aggregation to lead to the depressed growth of the 1st-layer AgNPs. In addition, when the reaction time was > 9 h, some dendrites formed on the top of the double-layer with a statistically uniform distribution (figure not shown). The dendrite layer could be regarded as a 3rd-layer although its coverage degree was low. The mean particle sizes in the 1st-layer and 2nd-layer are listed in Table 1.

**Table 1:** The mean sizes of the AgNPs in the double-layer Ag films prepared in 5/2.5 mM AgNO_3_/BuNH_2 _solution at different reaction times of 1.5-40 h

Reaction time (h)	Size of AgNPs (nm)	
	
	1st-layer	2nd-layer
1.5	20 ± 5	/
2	35 ± 10	/
5	40 ± 8	60 ± 15
18	35 ± 12	80 ± 20
25	45 ± 10	150 ± 30
40	50 ± 10	180 ± 30

The mean sizes were obtained by averaging the diameters of 15-50 particles in each layer

The time-dependent evolution of the microstructures of Ag/GL substrates discussed above was also observed when lower or higher AgNO_3_/BuNH_2 _concentrations were used. Note that almost no Ag dendrites were observed when the AgNO_3_/BuNH_2 _concentrations were 1/0.5 mM. The higher the AgNO_3_/BuNH_2 _concentrations were, the faster the Ag deposition occurred.

**Figure 2:**
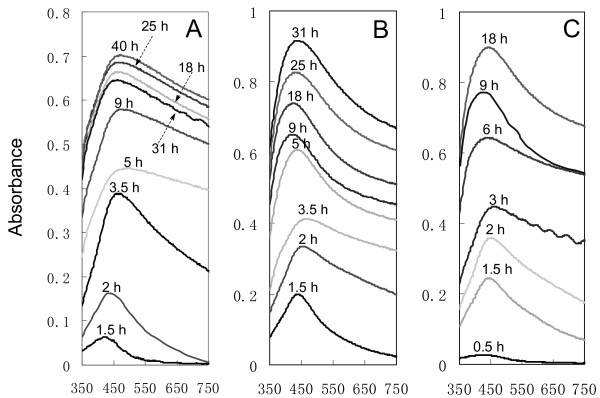
**UV-Vis absorption spectra of the AgNPs films prepared in AgNO_3_/BuNH_2 _ethanolic solutions at different reaction time**. The reactants concentrations: **(A) **1/0.5 mM, **(B) **5/2.5 mM, and **(C) **10/5 mM. The background spectra have been subtracted for all the UV-Vis absorption spectra.

**Figure 3:**
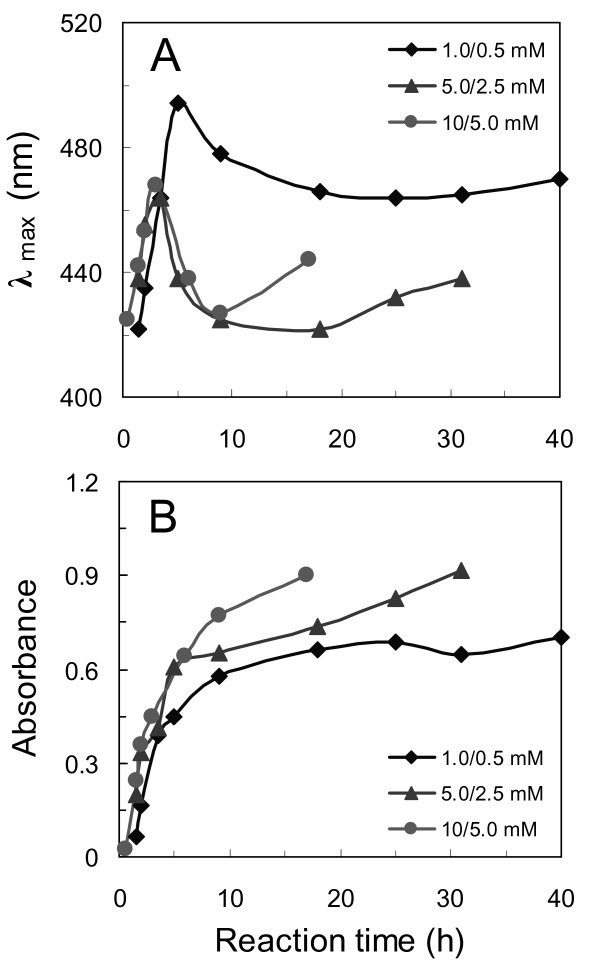
**The plots of the maximum absorption wavelength (λ_max_) and the absorbance at λ_max _of UV-Vis absorption spectra of the AgNPs films against the reaction time**. **(A) **λ_max _vs reaction time, and **(B) **absorbance at λ_max _vs reaction time.

The Ag nanofilms were found to exhibit strong plasmon absorption. Figure 2 shows the UV-Vis absorption spectra of the Ag films prepared at different reaction times for three different concentrations of reactants. It was found that the bandwidth of the plasmon resonance peak varied with the reaction time, especially for the 1/0.5 mM AgNO_3_/BuNH_2 _reaction solution. The plots of maximum absorption wavelength (λ_max_) and the absorbance at λ_max _against the reaction time are shown in Figure 3A,B, respectively. Figure 3A shows that λ_max _of the Ag films increased (shifted to longer wavelength) with the reaction time at the initial reaction stage, indicating that the size of AgNPs was increasing during this period [24,40]. It is interesting that when a maximum (1st turning point) was reached within 2-5 h, the λ_max _started to descend till a valley occurred (2nd turning point) followed by another increase. By comparison with the SEM images, we noticed that the 1st turning point was right around the reaction time when the 2nd-layer AgNPs started to appear. The formation of the 2nd-layer AgNPs and their growth in the direction normal to the substrate surface might lead to decrease in their diameter-to-height (*a/b*) ratio, and consequently a decrease in λ_max _[41]. The second rise in λ_max _was probably related to the formation of the 3rd-layer Ag dendrites. Since only few Ag dendrites were observed on the film prepared in the 1/0.5 mM AgNO_3_/BuNH_2 _solution, the second increase in λ_max _for these samples appeared to be much slower than others.

Unlike the undulatory variation of λ_max_, the absorbance at λ_max _of each UV-Vis spectrum increased consistently with reaction time as shown in Figure 3B. This means that the density of AgNPs or the thickness of the Ag films kept increasing throughout the reaction [42]. This increase exhibited a rapid kinetics at the initial reaction stage, followed by a relatively slow one. In short, the factors determining λ_max_, absorbance and bandwidth of the plasmon resonance are multifaceted, including the size, shape, and density of the metal particles, dispersion of particle sizes and their aggregation on the substrates [40-43]. Table 2 lists the maximum λ_max_, valley λ_max_, and the corresponding reaction time needed to reach them for the three reaction systems.

**Table 2:** The maximum λ_max_, valley λ_max_, maximum peak height of 780 cm^-^^1 ^band and the corresponding reaction time needed them to reach for the three reaction systems.

**AgNO**_**3**_**/BuNH**_**2**_**(mM/mM)**	**Maximum λ**_**max**_		**Valley λ**_**max**_		Maximum SERS	
	
	Value (nm)	Time (h)	Value (nm)	Time (h)	Peak height	Time (h)
1/0.5	494	5	464	25	4539	25
5/2.5	464	3.5	422	18	10831	18
10/5	468	3	427	9	10240	6

There may be two mechanisms in the formation process of the multilayer Ag nanofilms: (1) Ag^+ ^was reduced to AgNPs/nanostructures by BuNH_2 _directly on the glass surfaces; (2) AgNPs/nanostructures were formed in the ethanol and then assembled on the glass surfaces. When the 1/0.5 mM AgNO_3_/BuNH_2 _were used, the reaction solution did not change in color and remained transparent throughout the reaction. The UV-Vis spectra of the reaction solution were recorded at different reaction times and did not indicate a significant difference among them (data not shown). This could suggest that the AgNP double-layer film was formed directly on the substrates following the first mechanism: Ag^+ ^was first anchored on the negatively charged glass substrates (NaOH treated) followed by BuNH_2 _reduction to form AgNP seeds. Then more Ag^+ ^ions were reduced onto the seeds resulting in the AgNPs growth and the formation of the Ag double-layer film.

For the reaction solution of 5/2.5 mM AgNO_3_/BuNH_2_, the Ag double-layer film had been formed at approximately 5 h (see Figure 1c); while the color of the reaction mixture was found to start to become yellow at approximately 7 h. As the reaction time was prolonged, the color became darker and darker, and the transparency decreased gradually, suggesting that a bulk reduction took place and the AgNPs were formed in the reaction mixture. As time went on, the AgNPs aggregated and precipitated on the bottom of the reaction vessel and the surface of the Ag/GL substrates. A similar phenomenon was also observed for the 10/5 mM AgNO_3_/BuNH_2 _solution. Compared with the 1/0.5 mM AgNO_3_/BuNH_2 _solution in which almost no Ag dendrites were observed on the Ag double-layer film, the above-mentioned appearance was an indication that the formation of the 3rd-layer Ag dendrites possibly followed the second mechanism.

### SERS spectra of arsenate on Ag nanofilms

**Figure 4:**
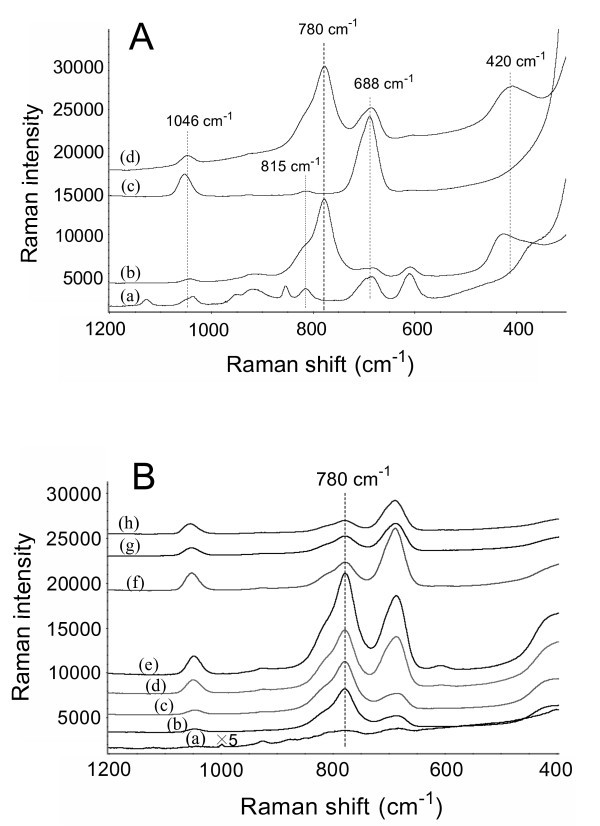
**Typical SERS spectra of arsenate using various Ag/GL substrates as active substrates**. **(A) **SERS spectra of arsenate: (a) 0 μg·l^-1 ^(background) and (b) 300 μg·l^-1 ^on Ag/GL-1/0.5-25 substrate; and (c) 0 μg·l^-1 ^(background) and (d) 250 μg·l^-1 ^on Ag/GL-5/2.5-18 substrate. **(B) **SERS spectra of 200 μg·l^-1 ^arsenate on various Ag/GL substrates prepared in 5/2.5 mM AgNO_3_/BuNH_2 _ethanolic solution at different reaction times: (a) 2 h, (b) 3.5 h, (c) 5 h, (d) 9 h, (e) 18 h, (f) 25 h, (g) 31 h, and (h) 40 h. The samples were air-dried before SERS measurements. The spectra were shifted vertically for clarity but the relative intensity was kept unchanged except for the curve a in (B).

Figure 4A shows typical background spectra (curves (a) and (c)) and SERS spectra of arsenate (curves (b) and (d)) on the Ag/GL-1/0.5-25 and Ag/GL-5/2.5-18 substrates, respectively. In the background spectrum (curve (a) in Figure 4A) of the Ag/GL-1/0.5-25 prepared in lower concentrations of AgNO_3_/BuNH_2_, there existed some Raman bands in the range of 300 to 1200 cm^-1^. Their intensities varied depending on the preparation conditions of the Ag films. When the higher concentrations of AgNO_3_/BuNH_2 _were used, the resulting Ag/GL-5/2.5-18 substrate had a simpler background spectrum (curve (c) in Figure 4A). Compared with the background spectrum of the Ag/GL-1/0.5-25, the intensities of two Raman bands centered at 1046 ± 8 cm^-1 ^and 688 ± 3 cm^-1 ^are much higher, while the other bands diminished or even disappeared.

In the SERS spectra of arsenate obtained on these two Ag films as shown in Figure 4A (curves (b) and (d)), most of the background bands could still be discerned but the peak intensities were suppressed. The two characteristic Raman bands of arsenate occurred at 780 ± 2 cm^-1 ^and 420 ± 10 cm^-1 ^due to the υ_1 _(A_1_) symmetric As-O stretch and a superposition of υ_2 _(A_1_) and υ_5 _(E) stretching modes of the arsenate molecule, respectively. Our recent study demonstrated that the υ_1 _(A_1_) symmetric As-O stretch resulted in a similar SERS band at approximately 780 cm^-1 ^using Ag nanofilms made using the mirror reaction [23]. Compared with the result obtained from the LB monolayers of AgNPs [38], this SERS band had a shift of approximately 20 cm^-1 ^to lower frequency. Since the 780 cm^-1 ^SERS band happened to be more intense than the 420 cm^-1 ^SERS band, and there were no serious interference bands around the 780 cm^-1 ^position in the background spectra, its intensity will be used to evaluate the SERS effects of arsenate in the subsequent studies. However, the arsenate SERS detection at low concentration using Ag/GL substrates may suffer from the interference of the nearest background band at approximately 815 cm^-1^.

### Optimization of Ag nanofilms for arsenate SERS sensing

**Figure 5:**
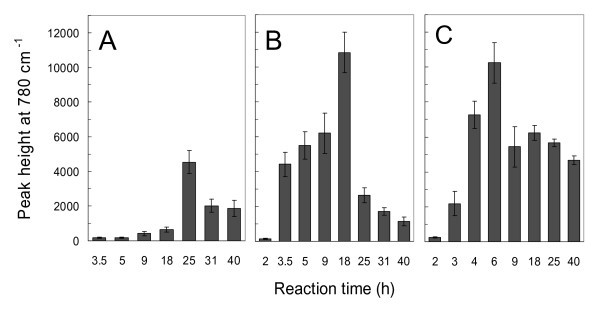
**Histograms indicating the change in peak heights of the 780 cm^-1 ^band with reaction time**. The peak heights were measured from the SERS spectra of 200 μg·l^-1 ^arsenate on the Ag/GL substrates prepared in **(A) **1/0.5 mM, **(B) **5/2.5 mM, and **(C) **10/5 mM AgNO_3_/BuNH_2 _ethanolic solutions with different reaction times.

Figure 4B shows the SERS spectra of arsenate (200 μg·l^-1^) on the Ag/GL substrates prepared in 5/2.5 mM AgNO_3_/BuNH_2 _solution at different reaction times of 2, 3.5, 5, 9, 18, 25, 31, and 40 h, respectively. It was of particular interest that the Ag/GL-5/2.5-18 as a SERS substrate gave the most intense arsenate peak among all eight Ag/GL substrates, while the peaks from Ag/GL-5/2.5-2 and Ag/GL-5/2.5-40 substrates were extremely weak. This clearly reflects the effect of the preparation conditions on the SERS sensitivity of the substrates. In order to more distinctly illustrate the results mentioned above, the peak heights of the arsenate Raman bands were plotted against the reaction time to yield a histogram (Figure 5B). From the figure, we can see that as the reaction time was prolonged from 2 to 40 h, the peak height increased gradually until a maximum appeared, and then decreased. The maximum peak height was obtained from the Ag/GL-5/2.5-18 substrate.

It is noticed that Ag/GL substrates covered by the AgNPs monolayer film resulted in much lower SERS enhancements than those covered by the AgNPs double-layer film (see Figures 1, 5B). When 2nd-layer AgNPs grew to 80 ± 20 nm in diameter (Ag/GL-5/2.5-18), the maximum SERS enhancement was observed for arsenate. The AgNPs with different sizes and shapes can have very different enhancement effects. Ag particles (or aggregates) with size of approximately 90 nm are reported to yield the highest SERS enhancement [39]. Moreover, as stated before, the aggregated and agglomerated AgNPs congeries with irregular shapes and structures had been formed in the Ag/GL-5/2.5-18. In this case, more "hotspots" or "active sites" could exist between two adjacent AgNPs or at the corners/edges of the irregular AgNPs in the double-layer film. The arsenate ions adsorbed on "hotspots" or "active sites" might produce extremely strong enhancements. It is possible that these two factors (suitable size of AgNPs and more "hotspots" or "active sites") contribute simultaneously to the remarkable SERS enhancement.

Similar reaction time-dependent profiles of SERS were observed for the Ag/GL substrates prepared in both lower (1/0.5 mM) and higher (10/5 mM) concentrations of AgNO_3_/BuNH_2 _solutions (Figure 5A,C). In summary, for a given concentration of AgNO_3_/BuNH_2 _concentration with constant molar ratio of 2:1, there was an optimum reaction time yielding a substrate with the maximum SERS enhancement. The higher the concentrations of the reactants were, the shorter the optimum reaction time was. In our experiments, the optimal Ag/GL substrate (i.e., Ag/GL-5/2.5-18) could be made in 5/2.5 mM AgNO_3_/BuNH_2 _ethanolic solution with a reaction time of 18 h at room temperature. The maximum peak height of the 780 cm^-1 ^band and the optimum reaction time for each set of AgNO_3_/BuNH_2 _concentrations are also listed in Table 2.

**Figure 6:**
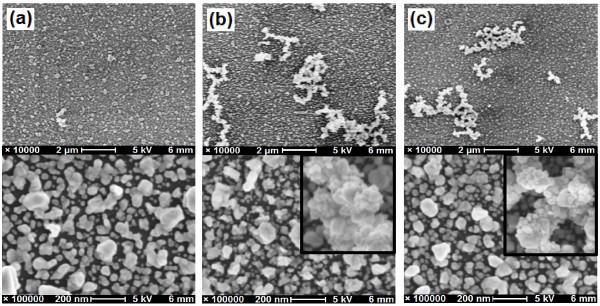
**SEM images of AgNPs multilayer films of the substrates**: **(a) **Ag/GL-1/0.5-25, **(b) **Ag/GL-5/2.5-18, and **(c) **Ag/GL-10/5-6. The top panels present the low magnification (large area) images, and the bottom panels are high magnification (high resolution) images indicating the nanostructures of Ag double-layer films. The insets in (b) and (c) show the high-resolution images of the corresponding Ag dendrites.

Comparing the results in Table 2, it is found that each optimum reaction time is equal to or near to the time needed to reach each valley λ_max _(2nd turning point in Figure 3A). It has been demonstrated that at the 2nd turning points, the 3rd-layer Ag dendrites had formed but still at the early stages for the two high concentrations of AgNO_3_/BuNH_2 _solutions. These observations imply that the 3rd-layer Ag dendrites may play a significant role in the arsenate SERS enhancements. Figure 6 presents the SEM images of the three optimized Ag/GL substrates (Ag/GL-1/0.5-25, Ag/GL-5/2.5-18, and Ag/GL-10/5-6) in both low magnification (large area) and high magnification (high resolution). The large area SEM images show that some Ag dendrites with 1-5 μm size appeared; larger area images (data not shown) show the Ag dendrites were distributed uniformly on the double-layer films of Ag/GL-5/2.5-18 and Ag/GL-10/5-6 substrates, while only few Ag dendrites were observed on the double-layer film of the Ag/GL-1/0.5-25 substrate. The high-resolution SEM images indicate that there is not much difference in AgNPs properties and microstructures among the three double-layer films except that the AgNPs look a little more irregular in the Ag/GL-5/2.5-18 substrate. The Ag dendrites consist of small AgNPs of 20-50 nm. The previous study also indicated that Ag dendrites are very SERS-active [19,22,44,45]. Therefore, the two Ag/GL substrates with Ag dendrites on them exhibited much higher SERS effects than that without Ag dendrites. For a given concentration of reactants, after the optimum reaction time, some AgNPs in the Ag dendrites agglomerated together to form big solid Ag masses (images not shown), leading to the decreasing SERS magnitude.

### Limit of detection of arsenate

**Figure 7:**
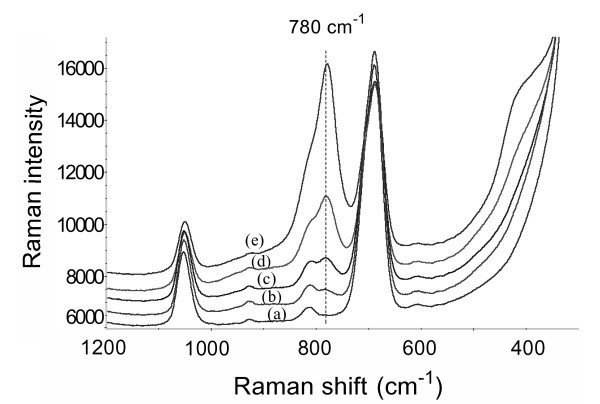
**SERS spectra of arsenate at the different concentrations using Ag/GL-5/2.5-18 as an active substrate: (a) 0, (b) 5, (c) 25, (d) 75, and (e) 150 μg·L^-1^**. The spectra were shifted vertically for clarity but the relative intensity was kept unchanged.

The SERS spectra of arsenate on every optimized substrate were measured in the concentration range 0-200 μg·l^-1^. Figure 7 shows the SERS spectra as a function of arsenate concentration recorded on Ag/GL-5/2.5-18 substrate. It is clear that a steady decrease in SERS intensity or peak height of the arsenate Raman band is observed with decreasing arsenate concentration. When the concentration is lower than 5 μg·l^-1^, the arsenate SERS band at approximately 780 cm^-1 ^appeared to be a shoulder of the 815 cm^-1 ^band, which is not easily discerned. Therefore, the LOD of Ag/GL-5/2.5-18 substrate for arsenate was determined to be approximately 5 μg·l^-1^. SERS sensing of low concentration arsenate may suffer from the interference of the 815 cm^-1 ^background Raman band. LODs of Ag/GL-1/0.5-25 substrate and Ag/GL-10/5-6 substrate were also determined, and the relatively high values are obtained, i.e., approximately 50 and 20 μg·l^-1^, respectively.

## Conclusions

A simple one-step electroless deposition process has been applied to prepare Ag/GL substrates for arsenate SERS sensing. Monolayer, double-layer, and multilayer AgNPs films with different nanostructural characteristics could be controllably deposited on glass by varying the reactant concentrations and deposition times. Two formation mechanisms have been proposed to lead to the multilayer Ag films. The SERS spectra of arsenate show that characteristic SERS bands of arsenate appear at approximately 780 and 420 cm^-1^, and the former possesses higher SERS intensity consistently regardless of the film nanostructures and the arsenate concentrations. By comparing the peak heights of the approximately 780 cm^-1 ^band of the SERS spectra, the most sensitive Ag/GL substrate for arsenate SERS sensing has been obtained in 5/2.5 mM AgNO_3_/BuNH_2 _solution with a deposition time of 18 h. This substrate is covered by a multilayer film consisting of one double-layer of AgNPs and one layer of Ag dendrites distributing uniformly over the double-layer. The lowest arsenate LOD was determined to be approximately 5 μg·l^-1 ^on this substrate, indicating its high SERS activity to arsenate.

The reproducibility, effects of coexisting electrolytes, and quantitative analyses of arsenate in spiked water samples and real groundwater samples using the optimal multilayer Ag nanofilm as the SERS-active substrate have been studied and the results will be reported in another article [46].

## Abbreviations

AAS: atomic absorption spectroscopy; AFS: atomic fluorescence spectroscopy; AgNPs: Ag nanoparticles; DI: deionized; FESEM: field-emission scanning electron microscope; ICP-AES or ICP-MS: inductively coupled plasma-atomic emission spectrometry or mass spectrometry; LB: Langmuir-Blodgett; LOD: limit of detection; MCL: maximum contaminant level; ppb: part per billion; SERS: surface-enhanced Raman scattering; WD: working distance; WHO: World Health Organization.

## Competing interests

The authors declare that they have no competing interests.

## Authors' contributions

JH and MH conceived of the study, carried out the preparation of multilayer Ag nanofilms, UV-Vis spectra measurements and SERS spectra collections, and drafted the manuscript. ZX participated in the SERS spectra analysis and discussion. JL participated in the SEM measurements. XM is the PI of the project participating in the design of the study and revised the manuscript, and conducted coordination. All authors read and approved the final manuscript.
